# Radiographic characteristics of rifampicin-resistant tuberculosis in the STREAM stage 1 trial and their influence on time to culture conversion in the short regimen

**DOI:** 10.1186/s12879-024-09039-z

**Published:** 2024-01-30

**Authors:** Chen-Yuan Chiang, Henry Bern, Ruth Goodall, Shun-Tien Chien, I. D. Rusen, Andrew Nunn, on behalf of the STREAM Sub-study Collaborators, Oumer Ali, Mekonnen Teferi, Muziwandile Ndlovu, Nosipho Ngubane, Rachel Bennet, Stella Fabiane, Sarah Meredith, Naranbat Nyamda, Bazarragchaa Tsogt, Phan-Thuong Dat, Pauline Howell, Meseret Hailu, Daniel Meressa, Samantha Aucock, Ronelle Moodliar

**Affiliations:** 1 Division of Pulmonary Medicine, Department of Internal Medicine, Wan Fang Hospital, Taipei Medical University, 111 Hsin-Long Road, Section 3, Taipei, 116 Taiwan; 2 Department of Internal Medicine, School of Medicine, College of Medicine, Taipei Medical University, 250, Wuxing St., Xinyi Dist., Taipei, 110 Taiwan; 3 MRC Clinical Trials Unit at UCL, London, UK; 4 Chest Hospital, Ministry of Health and Welfare, Tainan, Taiwan; 5 Vital Strategies, New York, USA

**Keywords:** Tuberculosis, HIV, Cavitation, Opacity, Culture conversion

## Abstract

**Background:**

Stage 1 of the STREAM trial demonstrated that the 9 month (Short) regimen developed in Bangladesh was non-inferior to the 20 month (Long) 2011 World Health Organization recommended regimen. We assess the association between HIV infection and radiographic manifestations of tuberculosis and factors associated with time to culture conversion in Stage 1 of the STREAM trial.

**Methods:**

Reading of chest radiographs was undertaken independently by two clinicians, and films with discordant reading were read by a third reader. Recording of abnormal opacity of the lung parenchyma included location (right upper, right lower, left upper, and left lower) and extent of disease (minimal, moderately-advanced, and far advanced). Time to culture conversion was defined as the number of days from initiation of treatment to the first of two consecutive negative culture results, and compared using the log-rank test, stratified by country. Cox proportional hazards models, stratified by country and adjusted for HIV status, were used to identify factors associated with culture conversion.

**Results:**

Of the 364 participants, all but one had an abnormal chest X-ray: 347 (95%) had opacities over upper lung fields, 318 (87%) had opacities over lower lung fields, 124 (34%) had far advanced pulmonary involvement, and 281 (77%) had cavitation. There was no significant association between HIV and locations of lung parenchymal opacities, extent of opacities, the presence of cavitation, and location of cavitation. Participants infected with HIV were significantly less likely to have the highest positivity grade (3+) of sputum culture (*p* = 0.035) as compared to participants not infected with HIV. Cavitation was significantly associated with high smear positivity grades (*p* < 0.001) and high culture positivity grades (*p* = 0.004) among all participants. Co-infection with HIV was associated with a shorter time to culture conversion (hazard ratio 1.59, 95% CI 1.05–2.40).

**Conclusions:**

Radiographic manifestations of tuberculosis among the HIV-infected in the era of anti-retroviral therapy may not differ from that among those who were not infected with HIV. Radiographic manifestations were not consistently associated with time to culture conversion, perhaps indicating that the Short regimen is sufficiently powerful in achieving sputum conversion across the spectrum of radiographic pulmonary involvements.

**Trial registration:**

ISRCTN ISRCTN78372190. Registered 14/10/2010. The date of first registration 10/02/2016.

**Supplementary Information:**

The online version contains supplementary material available at 10.1186/s12879-024-09039-z.

## Background

Two major obstacles in the successful management of tuberculosis (TB) are human immunodeficiency virus (HIV) coinfection and the emergence of multidrug/rifampicin -resistant tuberculosis (MDR/RR-TB). Until recently there has been very limited research into the choice of regimens for the treatment of MDR/RR-TB. STREAM was a randomized, non-inferiority phase 3 trial with the objective of evaluating a shortened regimen for MDR/RR-TB closely similar to that successfully used in Bangladesh in comparison to the locally used long MDR-TB regimen that followed 2011 World Health Organization (WHO) guidelines. The objective was to assess whether the shortened regimen was as effective as the long regimen. The results demonstrated that the 9 month (Short) regimen developed in Bangladesh [1] was non-inferior to the approximately 20 month (Long) regimen recommended by the WHO in 2011 [2, 3]. A favourable outcome was achieved in 78.8% and 79.8% of participants treated with the Short and Long regimens, respectively, a difference of 1.0% point (95% CI, − 7.5 to 9.5) [3].

The association between radiographic manifestations and outcomes of TB treatment has been previously investigated, but findings have not been consistent [4–7]. Studies have shown that clinical and radiographic manifestations of TB differed between those who were infected with HIV and those who were not [8–11], and the influence of HIV infection was associated with the level of CD4 [12–14]. Those who have a relatively low CD4 count are more likely to be smear negative and have atypical radiographic manifestations, including hilar and mediastinum lymphadenopathy, lower lung field TB, disseminated TB and pleural involvement. Clinical and radiographic manifestations of TB in those who have a relatively high CD4 count tend to be similar to those who are not infected with HIV.

One third of the participants enrolled in Stage 1 of the STREAM trial were HIV positive and an important question is to what extent the radiographic characteristics differ between these two population groups; and whether this might explain any difference observed in response to treatment. The objective of the present paper is to assess the association between HIV infection and radiographic manifestations of TB and factors associated with time to culture conversion in Stage 1 of the STREAM trial.

## Methods

### Study population

Between 2012 and 2015, MDR/RR-TB participants with and without HIV were enrolled in a phase 3 non-inferiority trial and randomised to either a 9 month (Short) regimen or the WHO-recommended 2011 (Long) regimen. Participants were 18 years of age or older with MDR/RR-TB who were sputum smear positive or, if co-infected with HIV, Xpert MTB/RIF assay (Cepheid, Sunnyvale, CA, United States) positive. Participants were ineligible if infected with a strain of *Mycobacterium tuberculosis* (Mtb) that was resistant to second-line injectables and/or a fluoroquinolone (as determined by line-probe assay). All patients in the short regimen group have been treated with a standardized regimen consisting of moxifloxacin, pyrazinamide, ethambutol, and clofazimine throughout, supplemented by kanamycin, high dose isoniazid and prothionamide in the initial intensive phase. Written informed consent was obtained from all participants. Full details are given in the main report of the study [3]. The trial was registered with ISRCTN on 14/10/2010 and has number ISRCTN78372190.

### Study procedures

Participants and clinicians were aware of individual treatment allocations; laboratory staff were not. Only the Independent Data Monitoring Committee and unblinded statisticians saw aggregate data by treatment arm during the trial. Participants had scheduled weekly visits for the first 4 weeks, then 4-weekly visits to 132 weeks for clinical review; sputum samples for smear and culture were collected at every visit from week 4. *Mycobacterium tuberculosis* isolates were sent to the study reference laboratory for drug susceptibility testing and strain genotyping to distinguish relapses from reinfections. Regular ECG monitoring was undertaken because of the risk of QT prolongation with a higher than standard dose of moxifloxacin.

### Chest radiograph reading and interpretation

All participants underwent a standard postero-anterior (PA) chest radiograph examination prior to enrolment. Reading of chest radiographs was undertaken independently by two clinicians unaware of the clinical and HIV status of study participants; radiographs classified as unreadable based on technical quality were excluded. Reading of the chest radiographs focused on lung parenchymal opacity and cavitation using published procedures [15]. Briefly, recording of abnormal opacity of the lung parenchyma included location (right upper, right lower, left upper, and left lower) and extent of disease (minimal, moderately-advanced, and far advanced). Both right and left lung parenchyma were divided into upper and lower lung field by a horizontal line across the mid-point of a vertical line from apex to diaphragm without taking the anatomy of the lung into consideration. Extent of disease was estimated by the sum of all areas of abnormality in which a boundary of abnormal opacity could be drawn. Minimal lesions were defined as an area less than that above a horizontal line across the 2nd chondrosternal conjunction of one lung; moderately-advanced lesions were defined as an area more than minimal lesions but less than one entire lung; far advanced lesions were defined as an area equivalent to or greater than one lung. Recording of cavitation included location (right upper, right lower, left upper, and left lower) and number (single or multiple). A cavity was defined as rounded lucency of at least one centimetre in diameter which could not be explained by overlapping structures (ribs, vessels or opacities) [15]. Agreement between the first and the second readers on the extent of opacities ranged from 81.5 to 91.5% and in reading cavities from 76.3 to 83.8%; Kappa for opacity reading ranged from 0.48 to 0.70 and for assessment of cavitation from 0.36 to 0.46 (Supplement). In the event of discordance between the two readers, films were read by a third, more senior pulmonologist. Films were read in batches (around 100 films) and discordant readings were discussed after each batch, aiming to reach consensus. All readers are internists certified in chest medicine (pulmonologists). They have been attending physicians for more than 20 years in Chest Hospital in Tainan, Taiwan.

### Outcomes

The main outcome of interest in this paper was time to culture conversion. Culture results obtained using acidified Ogawa (Kudoh medium) were used in analysis if available; results from Löwenstein-Jensen media were used if the Ogawa result was missing. Any culture result that was missing because the patient was no longer able to produce sputum was treated as a negative result, providing their last 2 available culture results (from sputum samples taken at separate visits) were negative. Smear positivity grade was defined as scanty if there were1–9 AFB per 100 fields, 1 + if 10–99 AFB per 100 fields, 2 + if on average 1–10 AFB per field (check at least 50 fields), and 3 + if on average more than 10 AFB per field (check at least 20 fields). Culture positivity grade was defined as 1 + if there were 10 ~ 100 colonies, 2 + if 100 ~ 200 colonies, and 3 + if more than 200 colonies; actual number of colony was recorded if 1–9 colonies. A culture for Mtb was considered positive even if only one colony was present and identified as Mtb. If an identification test result was not available for a particular culture, then for analysis purposes the culture was considered positive for Mtb provided that the colony growth appeared at least 14 days from the date of sputum processing and incubation.

Time to culture conversion was defined as the number of days from randomisation to the first of two consecutive negative culture results, collected on separate days. Participants who never achieved culture conversion were censored at the date of sputum collection that yielded their last culture result.

### Statistical analysis

Participants with culture-confirmed MDR/RR-TB, randomised to either the Short or Long regimen and included in the modified intention-to-treat trial population (mITT) with a readable chest radiograph were included in the analysis. Differences in HIV status according to baseline radiographic characteristics (extent of disease and presence or absence of cavitation) and sputum smear and culture positivity grades were assessed; the association between radiographic manifestations and sputum examination results (stratifying by HIV status) were investigated. Totals and proportions were tabulated and Fisher’s exact test used for comparison.

Time to culture conversion on the Short regimen was compared using the logrank test, stratified by country, and displayed using Kaplan Meier curves with respect to extent of lesions and presence of cavitation, presented separately for HIV positive and HIV negative participants (Supplementary material). Baseline radiographic and demographic characteristics (age, sex, BMI, diabetes, previous TB treatment, haemoglobin level, resistance to isoniazid, ethambutol or pyrazinamide) associated with time to culture conversion were analysed among participants on the Short regimen with a readable chest X-ray and at least one post-randomisation smear culture result. Cox proportional hazards models, stratified by country and adjusted for HIV status, were used to calculate hazard ratios of culture conversion according to these baseline characteristics; the best fitting multivariable model considering all variables except resistance to pyrazinamide (excluded due to the amount of missing data) was selected using Akaike Information Criteria (AIC) (limited to participants with complete data). The assumption of proportional hazards was tested based on the Schoenfeld residuals.

This analysis was initially restricted to participants randomised in South Africa and Ethiopia, as this population accounted for 98% of the HIV-positive cohort. The analysis was subsequently expanded to include participants randomised in Vietnam and Mongolia.

## Results

Between July 2012 and June 2015 a total of 689 participants were screened and 424 were randomized, 282 to the Short regimen and 142 to the Long regimen; 126 in Ethiopia, 33 in Mongolia, 165 in South Africa and 100 in Vietnam. 383 and 321 were included in the mITT and per protocol (PP) efficacy analyses respectively. Reasons for exclusion are described on the CONSORT flow chart (Fig. S1). Participants were followed-up for 132 weeks.

After excluding 16 participants without a chest X-ray, and 3 with a poor quality radiograph from the mITT population; there remained 364 participants, of which 118 (32.4%) were HIV positive. Of these 364 participants, all but one had an abnormal chest X-ray: 347 (95%) had opacities over upper lung fields, 318 (87%) had opacities over lower lung fields, 124 (34%) had far advanced pulmonary involvement, and 281 (77%) had cavitation. There was no significant association between HIV and locations of lung parenchymal opacities, extent of opacities, the presence of cavitation, location of cavitation, costophrenic obliteration, and pleural thickening (Table 1). Participants infected with HIV were significantly less likely to have the highest positivity grade (3+) of sputum culture (*p* = 0.035) as compared to participants not infected with HIV.

**Table 1:** Comparison of radiographic characteristics and baseline smear and culture results in the mITT population of the STREAM stage 1 trial according to HIV status

	Total *N* = 364		HIV Positive *N* = 118		HIV Negative *N* = 246		*P* value^1^
	N	Col %	N	Col %	N	Col %	
Upper opacity							
No	17	5	7	6	10	4	0.435
Yes	347	95	111	94	236	96	
Lower opacity							
No	46	13	13	11	33	13	0.614
Yes	318	87	105	89	213	87	
Right opacity							
No	39	11	14	12	25	10	0.718
Yes	325	89	104	88	221	90	
Left opacity							
No	34	9	7	6	27	11	0.177
Yes	330	91	111	94	219	89	
Isolated lower Opacity							
No	348	96	112	95	236	96	0.785
Yes	16	4	6	5	10	4	
Extent of opacity							
Nil/minimal/moderate	240	66	71	60	169	69	0.125
Far advanced	124	34	47	40	77	31	
Cavitation							
Absent	83	23	23	19	60	24	0.351
Present	281	77	95	81	186	76	
Location of cavities							
None	83	23	23	19	60	24	0.499
Unilateral	158	43	51	43	107	44	
Bilateral	123	34	44	37	79	32	
Costophrenic obliteration							
Absent	303	83	101	86	202	82	0.456
Present	61	17	17	14	44	18	
Pleural thickening							
Absent	348	96	112	95	236	96	0.785
Present	16	4	6	5	10	4	
Smear							
Negative/scanty/1+	117	32	44	37	73	30	0.110
2+	88	24	21	18	67	27	
3+	159	44	53	45	106	43	
Culture							
Negative/1–9/1+	83	23	28	24	55	22	**0.035**
2+	95	26	40	34	55	22	
3+	186	51	50	42	136	55	

^1^Fisher’s exact test

There is no evidence that extent of opacities was associated with smear or culture results in HIV-infected participants (Table 2). However, within the HIV negative cohort, the proportion with 3 + positive culture was lower among those with far advanced pulmonary involvement compared with participants with no, minimal, or moderate pulmonary involvement (*p* = 0.031). Cavitation was significantly associated with high smear positivity grades (*p* < 0.001) and high culture positivity grades (*p* = 0.004) among all participants, and remained so after the analysis was stratified by HIV status (Table 2).

**Table 2:** The association of radiographic manifestations and baseline smear and culture positivity in all the mITT population of the STREAM stage 1 trial and stratified by HIV status

	Total N (Col %)	Extent of opacity			Cavitation		
		Nil/minimal/moderate N (Col %)	Far advanced N (Col %)	*P* value^1^	No N (Col %)	Yes N (Col %)	*P* value^1^
HIV positive participants							
Participants	*N* = 118	*N* = 71	*N* = 47	-	*N* = 23	*N* = 95	-
Smear							
Negative/scanty/1+	44 (37)	30 (42)	14 (30)	0.358	16 (70)	28 (29)	0.002
2+	21 (18)	11 (15)	10 (21)		2 (9)	19 (20)	
3+	53 (45)	30 (42)	23 (49)		5 (22)	48 (51)	
Culture							
Negative/1–9/1+	28 (24)	15 (21)	13 (28)	0.292	10 (43)	18 (19)	0.023
2+	40 (34)	28 (39)	12 (26)		8 (35)	32 (34)	
3+	50 (42)	28 (39)	22 (47)		5 (22)	45 (47)	
HIV negative participants							
Participants	*N* = 246	*N* = 169	*N* = 77	-	*N* = 60	*N* = 186	-
Smear							
Negative/scanty/1+	73 (30)	51 (30)	22 (29)	0.364	22 (37)	51 (27)	0.003
2+	67 (27)	50 (30)	17 (22)		23 (38)	44 (24)	
3+	106 (43)	68 (40)	38 (49)		15 (25)	91 (49)	
Culture							
Negative/1–9/1+	55 (22)	38 (22)	17 (22)	0.031	18 (30)	37 (20)	0.043
2+	55 (22)	30 (18)	25 (32)		17 (28)	38 (20)	
3+	136 (55)	101 (60)	35 (45)		25 (42)	111 (60)	

^1^Fisher’s exact test

Of the 160 participants randomised in South Africa and Ethiopia to the Short regimen, four had no culture result post-randomisation and hence could not be included in a time to culture conversion analysis (Table 3); all except two of the remaining 156 participants achieved culture conversion.

**Table 3:** HIV-adjusted univariable & multivariable Cox regression models stratified by country (South Africa and Ethiopia)

Factor	Total	Univariable analysis		*P*-Value	Global *P*-Value	Multivariable analysis		*P*-Value	Global *P*-Value
		HR	95% CI			HR	95% CI		
HIV Status									
Negative	80	1		-	-	1		-	-
Positive	76	1.45 (0.99, 2.14)		0.059		1.59 (1.05, 2.40)		0.028	
Sex									
Male	85	1		-	-	1		-	-
Female	71	0.91 (0.66, 1.25)		0.561		0.78 (0.55, 1.10)		0.159	
Age									
18–24	36	1		-	0.560	-		-	-
25–34	65	0.93 (0.60, 1.45)		0.760		-		-	
35–44	30	0.77 (0.44, 1.33)		0.342		-		-	
45+	25	0.71 (0.41, 1.25)		0.241		-		-	
BMI									
< 16.0	17	1.62 (0.78, 3.38)		0.199	0.537	-		-	-
16.0–18.4	49	1.10 (0.60, 2.02)		0.754		-		-	
18.5–24.9	75	1.22 (0.68, 2.18)		0.511		-		-	
25.0+	15	1		-		-		-	
Smear result									
Negative/ scanty/ 1+	41	1.41 (0.95, 2.10)		0.090	0.195	-		-	-
2+	37	1.26 (0.85, 1.88)		0.255		-		-	
3+	78	1		-		-		-	
Culture result									
Negative/ 1–9 / 1+	18	1.85 (1.06, 3.22)		0.029	0.080	1.85 (1.00, 3.43)		0.050	0.089
2+	35	1.38 (0.86, 2.21)		0.185		1.52 (0.92, 2.53)		0.102	
3+	103	1		-		1		-	
Extent of opacities									
Minimal/moderate	104	1		-	-	-		-	-
Far advanced	52	0.93 (0.64, 1.33)		0.676		-		-	
Cavitation									
Absent	26	1		-	-	-		-	-
Present	130	0.88 (0.57, 1.37)		0.578		-		-	
Costophrenic obliteration									
Absent	127	1		-	-	-		-	-
Present	29	1.07 (0.70, 1.62)		0.753		-		-	
Pleural thickening									
Absent	150	1		-	-	-		-	-
Present	6	1.09 (0.48, 2.49)		0.832		-		-	
Diabetes									
No	147	1		-	-	-		-	-
Yes	9	1.18 (0.58, 2.40)		0.653		-		-	
Resistance to isoniazid									
No	10	1		-	-	-		-	-
Yes	138	0.63 (0.32, 1.23)		0.179		-		-	
Resistance to ethambutol									
No	40	1		-	-	-		-	-
Yes	108	0.78 (0.53, 1.14)		0.198		-		-	
Resistance to pyrazinamide									
No	45	1		-	-	-		-	-
Yes	85	0.90 (0.62, 1.30)		0.566		-		-	
Extent of cavities									
Nil	26	1		-	0.695	-		-	-
Unilateral	77	0.84 (0.53, 1.34)		0.468		-		-	
Bilateral	53	0.95 (0.58, 1.54)		0.832		-		-	
Previous treatment									
No	13	1		-	-	-		-	-
Yes	143	0.97 (0.54, 1.75)		0.922		-		-	
Haemoglobin									
< 11	35	1.25 (0.82, 1.89)		0.299	-	1.53 (0.98, 2.38)		0.060	-
11+	108	1		-		1		-	

Participants with low baseline culture positivity grades (negative, 1–9 or 1+) were more likely to achieve sputum culture conversion, compared to those with high positivity grade (3+, hazard ratio (HR) 1.85, 95% confidence interval (CI) 1.06–3.22). No baseline radiographic characteristic was significantly associated with time to culture conversion at the 10% level in the univariable analysis.

The multivariable model that yielded the lowest AIC (when adjusting for HIV status and stratifying by country) included baseline culture result, gender and haemoglobin levels. Co-infection with HIV and a baseline haemoglobin level below 11 g/dL were associated with a greater probability of culture conversion, with hazard ratios of 1.59 (95% CI − 1.05, 2.40) and 1.53 (95% CI − 0.98, 2.38), respectively. The model provided some support for women being less likely to culture convert compared to men (*p* = 0.159). Baseline culture result displayed a similar trend as for the univariable analysis.

The previous analysis was expanded to include participants randomised in Vietnam and Mongolia;　all except three of these 235 participants achieved culture conversion. The multivariable model that yields the lowest AIC for the expanded analysis is broadly consistent with the findings for the analysis restricted to South Africa and Ethiopia; HIV status and baseline culture result were associated with culture conversion. Additionally, the model provides some evidence that far advanced baseline opacities may be associated with being less likely to culture convert (HR − 0.76; 95% CI − 0.56, 1.03).

## Discussion

Previous studies have reported that HIV-infected TB patients with a low CD4 count were more likely to be smear negative [12]. In this study HIV-positive participants were significantly more likely to have negative or scanty smear, in part because of the enrolment criteria. Initially the enrolment criteria required participants to be smear positive regardless of their HIV status; however, with the introduction of version 5.0 of the protocol, participants infected with HIV could be enrolled if smear negative provided they were Xpert positive. The differential enrolment criteria with regards to HIV status likely also contributed to the difference in culture results. Patients with HIV infection were less likely to have 3 + culture results than patients not infected with HIV. However, the proportion of smear-positive TB cases among the HIV-infected participants of STREAM Stage 1 was substantially higher than the proportion reported in other studies [12, 13, 16], indicating that smear negative-culture positive pulmonary TB cases were under-representative in the STREAM trial.

Other studies have shown that radiographic manifestations of TB differ between those who are infected with HIV and those who are not, and that HIV-infected patients, especially those with low CD4 counts, are less likely to have extensive pulmonary involvement, and less likely to have cavitary lesions [12–14]. Immunosuppression is thought to be associated with rapid progression to symptomatic disease, leading to reduced bacterial load and cavitation at presentation [17]. CD4 counts were not systematically collected in stage 1 of the STREAM trial, but we did not find a significant association between HIV and locations of lung parenchymal opacities, extent of opacities, the presence of cavitation, location of cavitation, costophrenic obliteration, and pleural thickening, which may be because those participants who had HIV infection were likely to have had relatively high CD4 counts. The REMoxTB trial also reported that HIV had no significant effect on the radiological severity, and had an entry criterion of CD4 > 250 at baseline [18]. 

Different radiographic classification systems for TB have been developed for different purposes [19–24]. The method of reading applied in this study was adapted in 2008 from the Chest Radiograph Reading and Recording System (CRRS) developed in South Africa [21], aiming to standardize the classification of radiographic manifestations of pulmonary TB and to eliminate the dependence of individual readers. It has previously been used in a study of radiographic manifestations of pulmonary TB in diabetic patients in Taiwan [15]. The three readers in the present study participated in the diabetic study, thus were familiar with the methods. In the present study readers were blinded to clinical and HIV status of participants and the strength of agreement between the first two readers was moderate to substantial for the reading of opacity and cavity. Furthermore, radiographs for which there was any discordance were read by a third reader and the final result was determined by the majority result among the three readers followed by discussion to achieve consensus.

The association between cavitary lesion and higher bacillary load in pulmonary TB has been reported previously [18, 22, 25, 26]. We found that cavitation was significantly associated with high smear positivity grades and high culture positivity grades irrespective of HIV status. However, extent of opacities was not significantly associated with smear results or culture results, which may indicate that classification of extent of opacities into minimal, moderate and far-advanced is insufficiently discriminating. Recently Ralph et al. developed a numerical score for grading chest x-ray severity in smear positive pulmonary TB given by proportion of lung affected plus 40 in case of cavitation [22]. The REMoxTB trial applied Ralph’s numerical score and reported that the radiological severity of disease on chest x-ray prior to treatment in smear positive pulmonary TB patients was weakly associated with the bacterial burden, but this effect was lost in those without cavitation [18]. 

We found that HIV infection was associated with greater chance of culture conversion, in part because participants infected with HIV were significantly more likely to have negative or scanty smear, less likely to have 2 + positive smear, and significantly less likely to have the highest positivity grade (3+) of sputum culture as compared to participants not infected with HIV. Similarly, Telzak et al. reported that HIV infection is not associated with a longer time to clearance of sputum smears or cultures [27]. Additionally, HIV infection was not significantly associated with time to culture conversion among MDR-TB in Botswana [4], Tugela Ferry, South Africa [5], and Europe [28]. 

In this study, participants with low culture positivity grade had greater probability of culture conversion than those with high culture positivity grade. Similar findings have been reported previously. In a clinical trial in which moxifloxacin was substituted for isoniazid in a shortened regimen for the treatment of pulmonary TB, high sputum bacillary load on baseline smear was a negative predictor of week-8 culture negativity [29]. Holtz et al. reported that high initial sputum culture colony count was an independent predictor of a longer sputum culture conversion time among MDR-TB in Latvia [6]. 

In the present study, radiographic manifestations were not consistently associated with time to culture conversion, perhaps because the short regimen using high dose fluoroquinolone, high dose isoniazid, kanamycin and other 4 drugs in the intensive phase is sufficiently powerful in achieving sputum conversion across the spectrum of radiographic pulmonary involvements. The association between radiographic manifestations and time to culture conversion was not consistent across different studies [5, 6, 27, 29–32]. Telzak et al. reported that cavitary disease, numerous AFB on the initial smear, and no prior history of TB were independently associated with an increased number of days for both smear and culture conversion [27]. Clinical trials of substitution of moxifloxacin for isoniazid or ethambutol in drug-susceptible TB reported that cavitation was associated with a lower likelihood of culture conversion at week 8 [29] or 2 months [30], respectively. In MDR-TB, Holtz et al. reported that bilateral cavitation on chest radiography was a predictor of a longer sputum culture conversion time [6]; however, presence of cavitation was not associated with poor treatment outcome [7]. Yuen et al. reported that radiographically determined bilateral disease and cavitation were associated with a lower likelihood of sputum culture conversion [31]. In Ethiopia, alcohol drinking, higher sputum smear gradings, presence of cavitation, and consolidation were the determinants of time to sputum culture conversion among MDR-TB patients [32]. However, Brust et al. reported that in MDR-TB patients, presence of cavitation was not associated with longer time to conversion in Tugela Ferry, South Africa [5]. 

Anaemia was weakly associated with faster sputum conversion in this study. Previous studies have found anaemia common among the HIV-infected and TB patients [33], and associated with delayed sputum conversion [34], with one study even suggesting anaemia as a biomarker for severity of TB [35]. Isoniazid resistance was not associated with longer time to culture conversion in this study, perhaps because high dose moxifloxacin was used. A review by Donald and Diacon reported that in nearly all studies, the fluoroquinolones have shown bactericidal activity close to, or equal to, that of isoniazid [36]. Dorman et al. reported that substitution of moxifloxacin for isoniazid was associated with a small increase in Week-8 culture negativity [29]. In REMoxTB trial, patients treated with moxifloxacin in place of isoniazid in the standard regimen had faster conversion to culture negativity [37]. 

Strengths of this study included standardized reading of radiographic manifestations of TB by three readers thus not depending on an individual. A limitation was incomplete data on CD4 counts of the HIV-infected, and the enrolment criteria was slightly different according to HIV infection status. Furthermore, the prevalence of HIV infection was not balanced between countries, being very low in two of them.

In conclusion, radiographic manifestations of TB among the HIV-infected in the era of anti-retroviral therapy may not differ from that among those who were not infected with HIV. The HIV-infected were less likely to have a high culture positivity grade in part due to the enrolment criteria; they also had faster sputum conversion. Radiographic manifestations of TB were not consistently associated with time to sputum conversion in patients treated with the Short regimen, perhaps indicating that the Short regimen seems to be powerful in achieving sputum conversion across the spectrum of radiographic manifestations of TB. Those with high culture positivity grade having a higher bacillary load required longer time to achieve sputum conversion, supporting the recommendation of prolongation of the intensive phase of treatment in those with delayed smear conversion at 4 months [1].

### Supplementary Information

**Additional file 1.**

## Data Availability

Data collected for the study, including individual participant data and a data dictionary defining each field in the set, will be made available no later than 12 months after the end of the trial through the TBPACT data repository (https://c-path.org/programs/tb-pacts/). We will provide de-identified participant data, data dictionary, study protocol, a set of blank case record forms, and the informed consent form. Dr. Ruth Goodall should be contacted if someone wants to request the data from this study.

## Abbreviations

AIC: Akaike Information Criteria
CI: Confidence interval
CRRS: Chest Radiograph Reading and Recording System
HIV: Human immunodeficiency virus
HR: Hazard ratio
MDR/RR-TB: Multidrug/rifampicin -resistant tuberculosis
mITT: Modified intention-to-treat trial population
Mtb: *Mycobacterium tuberculosis*
PA: Postero-anterior
PP: Per protocol
STREAM: Evaluation of a Standardised Treatment Regimen of Anti-Tuberculosis Drugs for Patients with MDR-TB
WHO: World Health Organization
