# Supplementary material for: Radiographic characteristics of rifampicin-resistant tuberculosis in the STREAM stage 1 trial and their influence on time to culture conversion in the short regimen
Source: BMC Infect Dis. 2024 Jan 30;24:144. doi: 10.1186/s12879-024-09039-z (PMC10825976; doi:10.1186/s12879-024-09039-z)
Supplement: Supplementary file 1 — Additional file 1. [file 12879_2024_9039_MOESM1_ESM.docx]

Figure S1. Screening, Randomization, and Analysis of the Study Populations.

689 screened

424 randomised

**265 (38.5%) not randomised**

105 Not smear or GeneXpert positive

42 Rifampicin sensitive

34 Fluoroquinolone and/or injectable resistant

20 No informed consent

15 Unlikely to survive >4 months

13 Unsafe to enter study;

7 Rifampicin inconclusive

4 AST or ALT >5xULN

4 Unable to produce sputum

3 Fluorescein Diacetate (FDA) negative

18 Other, unspecified

142 randomised to receive Long regimen

282 randomised to receive Short regimen

**130 (91.5%) included in mITT analysis population**

**12 (8.5%) excluded**

7 no positive culture at baseline

2 rifampicin sensitive in reference laboratory

2 randomised in error

1 XDR-TB in reference laboratory

**253 (89.7%) included in mITT analysis population**

**29 (10.3%) excluded**

18 no positive culture at baseline

6 rifampicin sensitive in reference laboratory

4 randomised in error*

1 XDR-TB in reference laboratory

**6 (4.6%) not assessable for mITT primary analysis**

5 lost to follow-up after 76 weeks; culture negative when last seen

1 reinfection

**8 (3.2%) not assessable for mITT primary analysis**

1 lost to follow-up after 76 weeks; culture negative when last seen

7 reinfections

**87 (61.3%) included in PP analysis**

**12 (8.5%) excluded from mITT
 43 (30.3%) excluded from PP for not completing an adequate course of treatment.**

**234 (83.0%) included in PP analysis**

**29 (10.3%) excluded from mITT
 19 (6.7%) excluded from PP for not completing an adequate course of treatment.**

**4 (4.6%) not assessable for PP primary analysis**

3 lost to follow-up after 76 weeks; culture negative when last seen

1 reinfection

**7 (3.0%) not assessable for PP primary analysis**

1 lost to follow-up after 76 weeks; culture negative when last seen

6 reinfection

## Interventions

Medicines in the Short regimen were quality assured and supplied through The Union procurement mechanisms; supplies for the Long regimen were provided by the National Tuberculosis Programmes of participating countries.

### Short regimen

Drugs and doses by weight band in the Short regimen are shown below.

| **Product** | **Weight group** | | |
| --- | --- | --- | --- |
|  | Less than 33 kg | 33 kg to 50 kg | More than 50 kg |
| Moxifloxacin | 400 mg | 600 mg | 800 mg |
| Clofazimine | 50 mg | 100 mg | 100 mg |
| Ethambutol | 800 mg | 800 mg | 1200 mg |
| Pyrazinamide | 1000 mg | 1500 mg | 2000 mg |
| Isoniazid | 300 mg | 400 mg | 600 mg |
| Prothionamide | 250 mg | 500 mg | 750 mg |
| Kanamycin | 15 mg per kilogramme body weight (maximum 1g) | | |

All drugs were given in a single dosage daily (seven days a week) except for kanamycin which was given three times per week from week 12. Doses could be changed at the end of the intensive phase if participants had increased weight.

The intensive phase of the Short regimen could be extended from 16 to 20 or 24 weeks for participants whose smear has not converted by 16 or 20 weeks respectively.

Treatment that had been missed (up to 8 weeks in total) in either the intensive or the continuation phase could be made up by extending the relevant phase of the regimen by the number of days missed at the discretion of the treating clinician. Participants who missed more than 8 weeks in total were referred to the National TB Programme for further management but continued in follow-up to 132 weeks.

### Long regimen

Any modifications to the locally used standard of care regimen that was used as the Long regimen in the STREAM trial that occurred during the trial are also described below.

#### Mongolia

The intensive phase was composed of pyrazinamide (PZA), ethambutol (ETH), kanamycin (KAN), levofloxacin (LFX), ethionamide (ETO), and high-dose isoniazid (INH). This was given for at least 4 months after the participant first converted to sputum culture-negative, and for a minimum duration of 6 months.

The continuation phase was given for at least 12 months, to give a total treatment duration of 18 – 24 months, and was composed of PZA, ETH, LFX, and ETO.

#### South Africa

Prior to August 2015, the intensive phase was composed of ofloxacin (OFX), PZA, ETH, KAN or amikacin (AMK), ETO, and one of the following: terizidone (TZD), azithromycin (AZI), or clofazimine (CFZ). The continuation phase of treatment consisted of OFX, PZA, ETH, and TZD or CFZ.

After August 2015, the intensive phase of treatment was changed to moxifloxacin (MFX), PZA, TZD, ETO, and KAN; para-aminosalicylic acid (PAS) and INH could also be included based on drug sensitivity testing results. The continuation phase comprised PZA, MFX, TZD, and ETO.

The intensive phase was given for at least 4 months after the participant first converted to sputum culture-negative, and for a minimum duration of 6 months total. The continuation phase was given for 12 months, to make the total treatment duration at least 18 months.

#### Ethiopia

Prior to April 2013, the intensive phase was composed of LFX, PZA, ETH, capreomycin (CPM), ETO, and cycloserine (CYC). The continuation phase of treatment consisted of LFX, PZA, ETH, ETO, and CYC.

After April 2013, the intensive phase of treatment was changed to LFX, PZA, CPM, prothionamide (PTO), and CYC. The continuation phase comprised PZA, LFX, PTO, and CYC.

The intensive phase was given for at least 4 months after the participant first converted to sputum culture-negative, and for a minimum duration of 8 months total. The continuation phase was given for 10 months before April 2013 and 12 months after April 2013, to make the total treatment duration at least 18 months or 20 months, respectively.

#### Vietnam

The intensive phase was composed of PZA, ETH, KAN, LFX, PTO, and CYC. This was given for at least 4 months after the participant first converted to sputum culture-negative, and for a minimum duration of 6 months and a maximum duration of 10 months.

The continuation phase was given for 13 – 14 months after the completion of the intensive phase, and was composed of PZA, ETH, LFX, PTO and CYC.

## Full eligibility criteria

There were some minor modifications to the eligibility criteria during recruitment, full eligibility criteria with any changes are described here.

### Inclusion criteria

A participant will be eligible for entry to the study if he/she:

1. Is willing and able to give informed consent to be enrolled in the trial treatment and follow-up (signed or witnessed consent if the participant is illiterate)
2. Is aged 18 years or older
3. Has smear-positive pulmonary tuberculosis with initial laboratory result of resistance to rifampicin by line probe assay or other DST

- *changed to add ‘or is HIV positive and has GeneXpert-positive pulmonary tuberculosis’*
- *changed to ‘has an initial laboratory result of resistance to rifampicin by line probe assay (Hain Genotype LPA), GeneXpert or culture-based drug susceptibility testing (Version5.0, March 2013)’*

1. Is willing to have an HIV test and, if positive, is willing to be treated with ART in accordance with the national policies.
2. Agrees to use effective barrier contraception or have an intrauterine contraceptive device during treatment phase if a pre-menopausal woman
3. Has an identifiable address and expects to remain in the area for the duration of the study
4. Is willing to adhere to the follow-up schedule and to study procedures

### Exclusion criteria

A participant will not be eligible for entry to the study if he/she:

1. Is infected with a strain of *M. tuberculosis* resistant to a second-line injectable drug by line probe assay
2. Is infected with a strain of *M. tuberculosis* resistant to a fluoroquinolone by line probe assay
3. Has tuberculous meningitis or bone and joint tuberculosis
4. Is critically ill, and in the judgment of the investigator, unlikely to survive more than 4 months.
5. Is known to be pregnant or breast-feeding
6. Is unable to attend or comply with treatment or follow-up schedule
7. Is unable to take oral medication
8. Has AST or ALT >5 times the upper limit of normal
9. Has any condition (social or medical) which in the opinion of the investigator would make study participation unsafe.
10. Is taking any medications contraindicated with the medicines in either the trial or control regimen
11. Has a known allergy to any fluoroquinolone antibiotic
12. Is currently taking part in another trial of a medicinal product
13. Has a QTc interval of ≥500msec at screening

### Primary efficacy outcome

#### Favourable

A participant’s outcome will be classified as favourable if their last two culture results are negative unless they have previously been classified as unfavourable. These two cultures must be taken on separate visits (on different days); the latest of which being within the Week 132 window (that is no more than six weeks before 132 weeks since randomisation but with no upper bound).

Participants that don’t have a culture result within the Week 132 window because they were unable to produce sputum, will be classified as favourable if their last two cultures before the Week 132 window are negative and they have not previously been classified as unfavourable; such participants will be identified separately in tables.

#### Unfavourable

A participant’s outcome will be classified as unfavourable if:

1. They are discontinued from their allocated study treatment and subsequently restarted on a different MDR-TB regimen
2. Treatment is extended beyond the scheduled end of treatment for any reason other than making up of days when no treatment was given (missed treatment) for a maximum of eight weeks. A maximum of 14 days of extra treatment (irrespective of reason) is acceptable before it is classified as treatment extension. In addition, if the intensive phase of treatment has been extended for delayed sputum conversion (maximum 8-week extension permitted) the scheduled end of treatment will also be extended by the same amount, in accordance with Section 7.3.2 of the protocol.
3. They are restarted on any MDR-TB treatment after the scheduled end of treatment, but before 132 weeks after randomization.
4. They change their allocated study treatment for any reason other than (1) the replacement of a single drug or (2) for participants allocated to Regimen A when the change is as a result of changes in local guidelines and not related to any change in the participant’s circumstances or condition.
5. Bedaquiline is started where the allocated regimen did not originally contain that drug.
6. A drug from the class of nitroimidazoles is started
7. They die at any point during treatment or follow-up
8. At least one of their last two culture results, from specimens taken on separate occasions, is positive
9. They do not have a culture result within the Week 76 window or thereafter

Providing none of the other criteria above are met, starting a single drug is not considered to be a substantial change to the regimen and therefore does not result in an unfavourable outcome, with the exception of adding bedaquiline or a drug from the class of nitroimidazoles.

An extension of the intensive phase of treatment in any study arm does not constitute an unfavourable outcome, as long as the extension follows the protocol permitted algorithm for late smear conversion (Short regimen) or local policy (Long regimen).

Changes of treatment in participants allocated to Regimen A that result from a change in local guidelines not related in any way to any change in the participant’s circumstances or condition will not be classified as unfavourable. A sensitivity analysis will be conducted where these changes are classified as unfavourable.

All re-infections with a different strain are classified as not assessable.

A participant who has a culture result within the Week 76 window or thereafter, but not within the Week 132 window, having not otherwise been classified as unfavourable (based on the definitions above) will be regarded as not assessable and will be excluded from the primary analysis provided their last two cultures, from specimens taken on separate occasions, are negative. Such participants that don’t have a culture result within the Week 132 window because they were unable to produce sputum will be instead classified as favourable. Any participant who does not have a culture result within the Week 132 window and does not fulfil these criteria will be classified as unfavourable.

## Sample size assumptions

Randomization stratified by site and HIV-status was in a 2:1 ratio in favour of the Short regimen to collect more data on efficacy and safety on this regimen. Assuming 75% and 70% favourable outcomes on the Short and Long regimens respectively, 398 participants would be required to demonstrate non-inferiority with 80% power using a one-sided level of significance of 2.5% and allowing for up to 20% not assessable in a per-protocol (PP) analysis. A 10% margin of non-inferiority was considered an acceptable reduction in efficacy given the considerably reduced treatment duration

Based on the experience in Bangladesh and given that the trial population would include HIV-infected participants, not all of whom would be receiving anti-retroviral treatment (ART), it was assumed that the cure rate would be less than 88% reported by Van Deun and the Long regimen would perform better under trial conditions than the global mean of 48%. It was assumed that the proportion of favourable outcomes in the Short regimen would be marginally higher than in the Long regimen, 75% and 70% respectively. A 10% margin of non-inferiority was considered an acceptable reduction in efficacy given the considerably reduced pill burden and treatment duration. Based on a 2:1 allocation ratio in favour of the Short regimen, allowing for 20% of participants being classified as not assessable in a per-protocol analysis, a one-sided level of significance of 2.5% and 80% power, 398 participants would be required to demonstrate non-inferiority.

## Randomization Process

Separate randomization lists for each combination of strata were prepared by an independent statistician using permuted blocks of varying sizes. Participants were randomized using a web-based randomization system; if web access was not available at the time of randomization, a manual alternative using sealed envelopes was provided.

ADVERSE EVENTS

The proportions experiencing a grade 3 or higher adverse event were 48.2% (Short) and 45.4% (Long regimen), an adjusted difference of 2.8% (95% CI -7.3%, 12.9%). QT or QTcF prolongation to 500ms occurred more frequently on the Short regimen, 11.0% versus 6.4%, p=0.13.

Table S1: Baseline characteristics of modified intention to treat analysis population. Drug resistance is based on results from samples up to week 4 from central reference laboratory.

|  | | Long | Short | Total |
| --- | --- | --- | --- | --- |
| Total in MITT population | | **130** | **253** | 383 |
| Sex | Male | 83 (64%) | 151 (60%) | 234 (61%) |
| Age (years) | < 25 | 31 (24%) | 56 (22%) | 87 (23%) |
|  | 25 – 34 | 45 (35%) | 88 (35%) | 133 (35%) |
|  | 35 – 44 | 33 (25%) | 58 (23%) | 91 (24%) |
|  | ≥45 | 21 (16%) | 51 (20%) | 72 (19%) |
| Weight (kg) | < 33 | 0 | 1 (0%) | 1 (0%) |
|  | 33- 50 | 59 (45%) | 116 (46%) | 175 (46%) |
|  | ≥ 50 | 71 (55%) | 136 (54%) | 207 (54%) |
| BMI (kg/m^2^) | Median (IQR) | 19 (17 - 21) | 19 (17 - 21) | 19 (17 - 21) |
| HIV status | Positive | 40 (31%) | 85 (34%) | 125 (33%) |
| CD4 count (In HIV-infected)^1^ | Median (IQR) | 298 (166 - 532) | 239 (139 - 394) | 248 (143 - 429) |
| On ART at baseline (In HIV-infected) |  | 24 (60%) | 60 (71%) | 84 (67%) |
| Smoking status  N (%) | Never smoked | 82 (63%) | 164 (65%) | 246 (64%) |
|  | Ex-smoker | 36 (28%) | 59 (23%) | 95 (25%) |
|  | Current smoker | 12 (9%) | 30 (12%) | 42 (11%) |
| Smear | Negative | 3 (2%) | 8 (3%) | 11 (3%) |
|  | Positive | 127 (98%) | 245 (97%) | 372 (97%) |
| Previous TB treatment^2^ | None | 15 (12%) | 18 (7%) | 33 (9%) |
|  | Drug susceptible-TB treatment | 105 (81%) | 220 (87%) | 325 (85%) |
|  | Second-line treatment | 9 (7%) | 15 (6%) | 24 (6%) |
| Radiographic extent of disease^3^ | None or minimal | 14 (11%) | 28 (12%) | 42 (12%) |
|  | Moderate | 72 (58%) | 126 (53%) | 198 (54%) |
|  | Advanced | 39 (31%) | 85 (36%) | 124 (34%) |
| Radiographic extent of cavitation^3^ | None | 28 (22%) | 55 (23%) | 83 (23%) |
|  | Single | 13 (10%) | 34 (14%) | 47 (13%) |
|  | Multiple | 84 (67%) | 150 (63%) | 234 (64%) |
| Heart rate (bpm) | <75 | 21 (16%) | 52 (21%) | 73 (19%) |
|  | 75 – 99 | 70 (54%) | 125 (49%) | 195 (51%) |
|  | ≥100 | 39 (30%) | 76 (30%) | 115 (30%) |
| Fredericia corrected QTcF (ms) | < 400 | 58 (45%) | 112 (44%) | 170 (44%) |
|  | 400 – 449 | 71 (55%) | 136 (54%) | 207 (54%) |
|  | 450 - 499 | 1 (1%) | 5 (2%) | 6 (2%) |
| Drug resistance to: | Isoniazid^4^ | 118 (93%) | 234 (94%) | 352 (94%) |
|  | Ofloxacin^5^ | 3 (3%) | 2 (1%) | 5 (1%) |
|  | Kanamycin or capreomycin^5^ | 1 (1%) | 3 (1%) | 4 (1%) |
|  | Pyrazinamide^6^ | 58 (59%) | 130 (63%) | 188 (62%) |

^1^ 52 HIV-infected participants were missing CD4 count (13 Long regimen, 39, Short regimen)

^2^ 1 participant on the Short regimen had no previous treatment recorded

^3^ 19 participants had no assessable chest x-ray readings (5 Long regimen,14 Short regimen)

^4^ Based on 127 on Long regimen and 248 on Short regimen with results available.

^5^ Based on 120 on Long regimen and 237 on Short regimen with results available.

^6^ Based on 99 on Long regimen and 206 on Short regimen with results available

Comparison of chest radiograph readings by reader 1 and reader 2 in the STREAM stage I trial

1. Right upper opacities

| Reader 2

Reader 1 | no yes | Total

-----------+----------------------+----------

no | 52 14 | 66

| 78.79 21.21 | 100.00

-----------+----------------------+----------

yes | 20 314 | 334

| 5.99 94.01 | 100.00

-----------+----------------------+----------

Total | 72 328 | 400

| 18.00 82.00 | 100.00

Expected

Agreement Agreement Kappa Std. Err. Z Prob>Z

-----------------------------------------------------------------

91.50% 71.44% 0.7024 0.0499 14.07 <0.0001

1. Right lower opacities

| Reader 2

Reader 1 | no yes | Total

-----------+----------------------+----------

0 | 82 32 | 114

| 71.93 28.07 | 100.00

-----------+----------------------+----------

1 | 38 248 | 286

| 13.29 86.71 | 100.00

-----------+----------------------+----------

Total | 120 280 | 400

| 30.00 70.00 | 100.00

Expected

Agreement Agreement Kappa Std. Err. Z Prob>Z

-----------------------------------------------------------------

82.50% 58.60% 0.5773 0.0500 11.55 0.0000

1. Left upper opacities

| Reader 2

Reader 1 | no yes | Total

-----------+----------------------+----------

0 | 47 9 | 56

| 83.93 16.07 | 100.00

-----------+----------------------+----------

1 | 25 319 | 344

| 7.27 92.73 | 100.00

-----------+----------------------+----------

Total | 72 328 | 400

| 18.00 82.00 | 100.00

Expected

Agreement Agreement Kappa Std. Err. Z Prob>Z

-----------------------------------------------------------------

91.50% 73.04% 0.6847 0.0494 13.85 0.0000

1. Left lower opacities

| Reader 2

Reader 1 | no yes | Total

-----------+----------------------+----------

0 | 55 30 | 85

| 64.71 35.29 | 100.00

-----------+----------------------+----------

1 | 44 271 | 315

| 13.97 86.03 | 100.00

-----------+----------------------+----------

Total | 99 301 | 400

| 24.75 75.25 | 100.00

Expected

Agreement Agreement Kappa Std. Err. Z Prob>Z

-----------------------------------------------------------------

81.50% 64.52% 0.4786 0.0498 9.62 0.0000

1. Extent of opacities

| Reader 2

Reader 1 | None Minimal Moderate Far-advanced| Total

-----------+--------------------------------------------+----------

None | 1 1 0 0 | 2

| 50.00 50.00 0.00 0.00 | 100.00

-----------+--------------------------------------------+----------

Minimal | 1 34 53 1 | 89

| 1.12 38.20 59.55 1.12 | 100.00

-----------+--------------------------------------------+----------

Moderate | 0 9 124 17 | 150

| 0.00 6.00 82.67 11.33 | 100.00

-----------+--------------------------------------------+----------

Far | 1 0 50 108 | 159

advanced | 0.63 0.00 31.45 67.92 | 100.00

-----------+--------------------------------------------+----------

Total | 3 44 227 126 | 400

| 0.75 11.00 56.75 31.50 | 100.00

Expected

Agreement Agreement Kappa Std. Err. Z Prob>Z

-----------------------------------------------------------------

66.75% 36.25% 0.4784 0.0347 13.78 0.0000

1. Right upper cavities

| Reader 2

Reader 1 | no yes | Total

-----------+----------------------+----------

0 | 161 52 | 213

| 75.59 24.41 | 100.00

-----------+----------------------+----------

1 | 43 144 | 187

| 22.99 77.01 | 100.00

-----------+----------------------+----------

Total | 204 196 | 400

| 51.00 49.00 | 100.00

Expected

Agreement Agreement Kappa Std. Err. Z Prob>Z

-----------------------------------------------------------------

76.25% 50.07% 0.5244 0.0499 10.50 0.0000

1. Right lower cavities

| Reader 2

Reader 1 | no yes | Total

-----------+----------------------+----------

0 | 304 19 | 323

| 94.12 5.88 | 100.00

-----------+----------------------+----------

1 | 46 31 | 77

| 59.74 40.26 | 100.00

-----------+----------------------+----------

Total | 350 50 | 400

| 87.50 12.50 | 100.00

Expected

Agreement Agreement Kappa Std. Err. Z Prob>Z

-----------------------------------------------------------------

83.75% 73.06% 0.3968 0.0484 8.20 0.0000

1. Left upper cavities

| Reader 2

Reader 1 | no yes | Total

-----------+----------------------+----------

0 | 186 39 | 225

| 82.67 17.33 | 100.00

-----------+----------------------+----------

1 | 32 143 | 175

| 18.29 81.71 | 100.00

-----------+----------------------+----------

Total | 218 182 | 400

| 54.50 45.50 | 100.00

Expected

Agreement Agreement Kappa Std. Err. Z Prob>Z

-----------------------------------------------------------------

82.25% 50.56% 0.6410 0.0500 12.83 0.0000

1. Left lower cavities

| Reader 2

Reader 1 | no yes | Total

-----------+----------------------+----------

0 | 276 25 | 301

| 91.69 8.31 | 100.00

-----------+----------------------+----------

1 | 59 40 | 99

| 59.60 40.40 | 100.00

-----------+----------------------+----------

Total | 335 65 | 400

| 83.75 16.25 | 100.00

Expected

Agreement Agreement Kappa Std. Err. Z Prob>Z

-----------------------------------------------------------------

79.00% 67.04% 0.3628 0.0483 7.51 0.0000

1. Number of cavities

| Reader 2

Reader 1 | None Single Multiple | Total

-----------+---------------------------------+----------

None | 70 19 47 | 136

| 51.47 13.97 34.56 | 100.00

-----------+---------------------------------+----------

Single | 4 23 10 | 37

| 10.81 62.16 27.03 | 100.00

-----------+---------------------------------+----------

Multiple | 23 30 172 | 225

| 10.22 13.33 76.44 | 100.00

-----------+---------------------------------+----------

Total | 97 72 229 | 398

| 24.37 18.09 57.54 | 100.00

Expected

Agreement Agreement Kappa Std. Err. Z Prob>Z

-----------------------------------------------------------------

66.58% 42.54% 0.4185 0.0372 11.25 0.0000

1. Costophrenic obliteration

| Reader 2

Reader 1 | None Right Left Both | Total

-----------+--------------------------------------------+----------

None | 312 24 29 2 | 367

| 85.01 6.54 7.90 0.54 | 100.00

-----------+--------------------------------------------+----------

Right | 1 14 0 1 | 16

| 6.25 87.50 0.00 6.25 | 100.00

-----------+--------------------------------------------+----------

Left | 4 0 10 0 | 14

| 28.57 0.00 71.43 0.00 | 100.00

-----------+--------------------------------------------+----------

Both | 1 0 0 2 | 3

| 33.33 0.00 0.00 66.67 | 100.00

-----------+--------------------------------------------+----------

Total | 318 38 39 5 | 400

| 79.50 9.50 9.75 1.25 | 100.00

Expected

Agreement Agreement Kappa Std. Err. Z Prob>Z

-----------------------------------------------------------------

84.50% 73.67% 0.4113 0.0326 12.63 0.0000

1. Plueral thickening

| Reader 2

Reader 1 | None Left Right Both | Total

-----------+--------------------------------------------+----------

None | 369 12 9 0 | 390

| 94.62 3.08 2.31 0.00 | 100.00

-----------+--------------------------------------------+----------

Left | 1 3 0 0 | 4

| 25.00 75.00 0.00 0.00 | 100.00

-----------+--------------------------------------------+----------

Right | 2 0 3 0 | 5

| 40.00 0.00 60.00 0.00 | 100.00

-----------+--------------------------------------------+----------

Both | 0 0 0 1 | 1

| 0.00 0.00 0.00 100.00 | 100.00

-----------+--------------------------------------------+----------

Total | 372 15 12 1 | 400

| 93.00 3.75 3.00 0.25 | 100.00

Expected

Agreement Agreement Kappa Std. Err. Z Prob>Z

-----------------------------------------------------------------

94.00% 90.75% 0.3513 0.0328 10.71 0.0000
